# Bioelectrical Impedance Analysis for Monitoring Fluid and Body Cell Mass Changes in Patients Undergoing Cardiopulmonary Bypass

**DOI:** 10.21470/1678-9741-2019-0152

**Published:** 2020

**Authors:** Mustafa Göz, Cemil Sert, Abdussamet Hazar, Mehmet Salih Aydın, Nazim Kankılıç

**Affiliations:** 1 Department of Cardiovascular Surgery, Medical School of Harran University, Şanliurfa, Turkey.; 2 Department of Biophysics, Medical School of Harran University, Şanliurfa, Turkey.; 3 Department of Cardiovascular Surgery, Malatya Education and Research Hospital, Malatya, Turkey.

**Keywords:** Bioelectrical Impedance, Fat Mass, Lean Body Mass, Total Body Water, Cardiopulmonary Bypass

## Abstract

**Objective:**

To evaluate preoperative and postoperative body fluid distribution with a bioelectrical impedance analyzer in patients undergoing cardiopulmonary bypass.

**Methods:**

Fifteen adult patients undergoing cardiopulmonary bypass were included in this study. Total body fluid changes, basal metabolism rates, body fat masses, lean body masses, and total cell masses were recorded. The patients’ values were measured before anesthesia, after anesthesia, after sternotomy, at the 5^th^, 30^th^, and 60^th^ minutes of cardiopulmonary bypass, and on the 1^st^, 3^rd^, and 5^th^ postoperative days. All values were compared with preoperative values.

**Results:**

Total body fluid changed significantly after cardiopulmonary bypass (*P*<0.01). Metabolic velocity significantly changed compared to preoperative measurements (*P*<0.05). Fat mass and lean body mass also changed significantly. Body mass index and phase angle did not change significantly (*P*>0.05).

**Conclusion:**

Changes in body fluids during and after cardiopulmonary bypass are inevitable. The increase in total body weight shows that this fluid load shifts to the extracellular space during bypass and the fluid load in this area passes into the intravascular area in the early postoperative period. This may cause edema and dysfunction in the major organs. Therefore, the fluid balance should be adjusted very carefully, especially during the bypass phase and the early postoperative period.

**Table:** 

Abbreviations, acronyms & symbols	
AVR	= Aortic valve replacement
BIA	= Bioelectrical impedance analysis
BMI	= Body mass index
BMR	= Basal metabolic rate
CABG	= Coronary artery bypass grafting
CCT	= Cross-clamp time
CPBT	= Cardiopulmonary bypass time
FM	= Fat mass
ICU	= Intensive care unit
LBM	= Lean body mass
MVR	= Mitral valve replacement
SPSS	= Statistical Package for the Social Sciences
TBW	= Total body water

## INTRODUCTION

Cardiac surgery with cardiopulmonary bypass leads to increase in total body fluid due to the prime solution and intravenous fluids. Additionally, interaction of extracorporeal circulation and prime volume with the body fluids causes a systematic inflammation^[1]^. Thus, the water retention in vital areas appears to cause notable clinical symptoms^[1]^. Therefore, perioperative fluid flow observation is closely related to prediction of morbidity.

Bioelectrical impedance analysis (BIA) is a non-invasive method to determine body fluid distribution^[2]^. This technique involves releasing a very little current in the tissues^[2]^. In a clinic, a single frequency of 50 kHz is used to make BIA measurements. These high-frequency electrical currents pass through both intra and extracellular fluids to calculate total body water (TBW). However, currents < 5 kHz of frequency proceed through the extracellular space and provide clinically useful information regarding circulation changes. Despite the use of BIA in medical sciences for a long while as a cost effective and relatively easy way of assessing the changes in body fluids, there are a quite limited amount of experiences regarding the use of BIA in cardiac surgery^[3-7]^.

Cardiac surgery with cardiopulmonary bypass is interrelated with a high load of fluids that causes an increase in total body fluids due to the pump solution and intravenous fluids^[3]^. Thus, it causes reduction of both hemoglobin and hemodilution^[3]^. Following the cardiac surgery, patients are usually given diuretic preparation to lower the TBW^[3]^. These are clinically kept track of.

In this study, body fluid changes, basic metabolic rate, body cell mass, fat-free body mass, and fat mass (FM) changes are studied before, during, and after the cardiac surgery by using BIA.

## METHODS

### Choosing Patients

After approved by the Clinic Committee of Ethics, 15 patients who had undergone adult open cardiovascular surgery were included in the study. Patients with systemic inflammatory disease, infection, recurrent cardiac surgery, overweight (body mass index [BMI] > 30), underweight (BMI < 18,5), and very low ejection fraction (≤ 30%) were excluded from the study.

### Anesthesia and Cardiopulmonary Bypass Technique

In order to help to control the high blood pressure and tachycardia before the surgery, 0.05-0.1 mg/kg of intravenous midazolam was applied. In induction, cases were endotracheally intubated after gaining enough amount of muscular relaxation with 3-10 mg/kg of fentanyl, 0.2-0.3 mg/kg of etomidate, and 0.6 mg/kg of rocuronium. Endotracheal intubation and anesthesia were performed in supine position. Then, central venous catheterization and left radial artery monitoring were performed. Nasopharyngeal and rectal heat probes were applied. During anesthesia, 5-channel electrocardiogram and blood oxygen saturation and end-tidal carbon dioxide monitoring were performed.

Dideco Compac Flo (Sorin Group, Italy) membrane oxygenator and Sasan Containerless Tubing Set (Sasan Medical Supplies Inc., Ankara, Turkey) were used in cardiopulmonary bypass. As the prime solution, 1200 cc of Lactated Ringer, 100 cc of 20%Mannitol, 20 cc of NaHCo3, 1cc of heparin, and 1g of cefazolin were used. All the cases were kept in mild hypothermia (26-32ºC). Perfusion pressure was kept control of (60-70 mmHg). Full cardiac flow was maintained in 2,4l/m^2^. Throughout the operation, activated clotting time was kept > 480 sec. Blood gas analyses were checked with alphastat mechanism. Until all the cases’ rectal heat levels reached 37,5ºC, they were heated by keeping a 10ºC gradient between water and blood heat. Immediately after reaching adequate cardiac performance, the operation was terminated by exiting the cardiopulmonary bypass.

### Tracking the Body Fluids and BIA Measurements

A Biodynamic P450 Bioelectrical Impedance Analyzer (Biodynamics Corporation, Washington, USA) was utilized before, during, and five days after the cardiopulmonary bypass in order to study and evaluate the fluid change, basic metabolic pace, body FM, and total cell mass. All patients' hands and feet were cleared for measurements. Two electrodes were connected to the right hand and right foot. Stimulating electrodes were attached to the dorsal sides of the second and third metacarpal and metatarsal joints. The recording electrodes were connected to the dorsal sides of the hands and feet. The patients received a current of 500uA. The current was too small to be felt by the patients. Measurements were made before, during, and after anesthesia, after sternotomy, at the 5^th^, 30^th^, and 60^th^ minutes of cardiopulmonary bypass, and on the1^st^, 3^rd^,and 5^th^ postoperative days. Daily fluid balance was monitored in all cases until the preoperative period and the postoperative 5^th^ day.

### Statistical Analysis

The Statistical Package for the Social Sciences software (SPSS, Chicago, Illinois, USA), version 11.5, was used for statistical analysis. The measurements were compared with preoperative values by using Wilcoxon test and statistical analyses were performed. *P*-values < 0.05 were considered significant.

## RESULTS

The patients’ general characteristics are shown in Table 1.

**Table 1:** Patients' general characteristics.

Age (years)	58±4.59
Males (n [%])	9 (%60)
Females (n [%])	6 (%40)
Ejection fraction (%)	46.26±7.48
CCT (min)	64.7±31.1
CPBT (min)	108.3±24.6
ICU stay (days)	3.21±1.01
Hospital stay (days)	8.4±4.21
CABG (n [%])	8 (%53,3)
MVR (n [%])	3 (%20)
AVR (n [%])	4 (%26,6)

AVR=aortic valve replacement; CABG=coronary artery bypass grafting; CCT=cross-clamp time; CPBT=cardiopulmonary bypass time; ICU=ıntensive care unit; MVR=mitral valve replacement

**Fig. 1:**
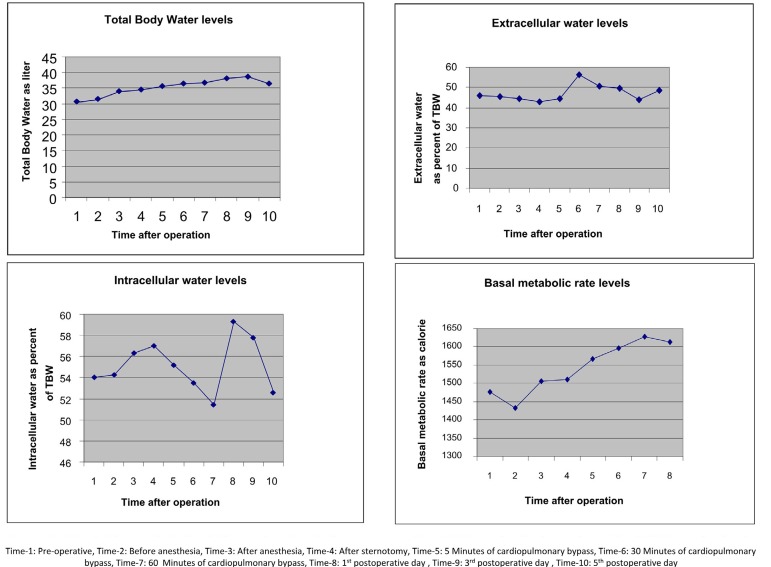
Changes in total body water (TBW), extracellular water, intracellular water, and basal metabolic rate levels after cardiopulmonary bypass.

TBW showed statistically significant changes in the post-bypass stage (*P*<0.01) (Figure 1). However, the amount of extracellular and intracellular fluids did not change significantly. The amount of extracellular fluid increased 60 minutes after bypass and one day after bypass, and then returned to normal (Figure1). The level of intracellular fluid also increased on the 1^st^ and 3^rd^ days after bypass and returned to normal on the 5^th^ postoperative day (Figure 1).

**Fig. 2:**
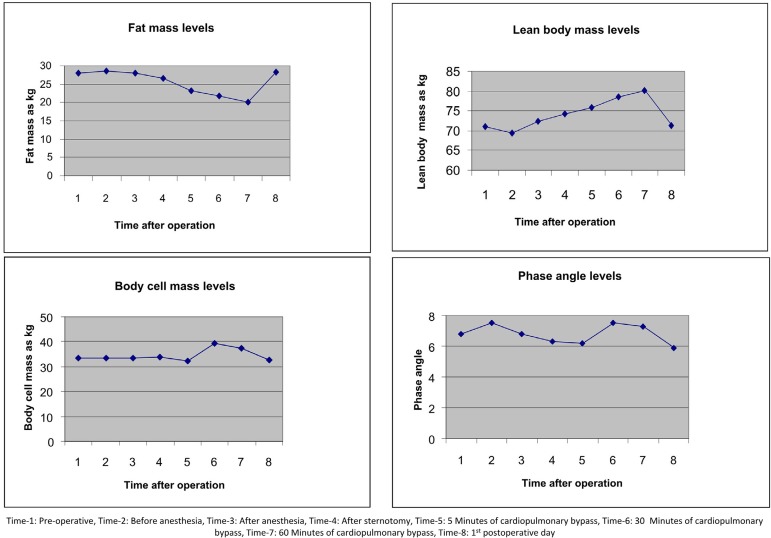
Changes in fat mass, lean body mass, body cell mass, and phase angle levels after cardiopulmonary bypass.

Basal metabolic rate (BMR) significantly changed compared to preoperative measurements (*P*<0.05). This change was bigger after the 60-minute post-bypass measurements (*P*<0.01) (Figure 1). FM changed significantly (*P*<0.05) (Figure 2). Lean body mass (LBM) also changed significantly (*P*<0.05). This change started after the 5^th^ minute post-bypass and returned to normal on the 1^st^ postoperative day (Figure 2). Total cell mass changed significantly. This change occurred after the 5^th^ minute post-bypass measurements (Figure 2). BMI and phase angle did not change significantly (*P*>0.05) (Figure 2).

## DISCUSSION

It is observed that total body fluid increases significantly after cardiac surgery. However, it is an important problem when this increase of fluid starts exactly after the operation and when it returns to normal. It is also important how this is reflected in the extracellular and intracellular fluid changes and how BMR and body composition values change after cardiac surgery. Studies on this subject are very limited. Since we could not find a detailed study, we recorded the measurements up to the 5^th^ day and our study was made detailed. Significant correlations between various body composition methods, such as isotope dilution^[8]^, total-body potassium count^[9]^, dual radiographic absorptiometry^[10,11]^, or densitometry^[12]^, have been shown. BIA is a body composition measurement method. It is used in many ways and in many disease groups^[13-15]^. BIA can be easily performed to determine fat-free mass, fluid deposition, peritoneal or hemodialysis fluid exchange, perioperative fluid deposition, and body weight in weak, obese, and sick people^[16-18]^.

David Bracco et al.^[19]^ measured body FM and body fluid mass before and after the operation in 22 patients undergoing cardiopulmonary bypass. They observed an increase of 8-10% in fat-free mass. They also observed an increase in fluid mass. The first mechanism is that it triggered edema formation due to surgical translocation and tissue trauma. The second mechanism is macro and microcirculation changes caused by intestinal permeability changes^[20]^. Fiogbé E et al.^[21]^investigated the effect of water aerobic exercise training on heart rate autonomic modulation and body composition in the rehabilitation of patients with coronary artery disease. In BIA evaluation, it was observed that body compositions did not change with disease or rehabilitation. Contrary to that study, this change is seen in patients who underwent cardiovascular surgery in our study.

It is important to measure the amount of intracellular and extracellular fluids separately in BIA, because intracellular edema may be an indicator of organ dysfunction and may lead to organ failure^[22,23]^. In recent studies, it was emphasized that BIA may be useful in determining the prognosis and treatment of patients with heart failure^[23-25]^. It is not possible to detect this by methods other than BIA. In our study, intracellular fluid showed no significant change. However, it was different during the follow-up period. Intracellular fluid returned to baseline at the 5^th^ postoperative day. The amount of extracellular fluid did not change significantly, but it slightly increased. Compared to the preoperative measurements, it showed a significant increase in the measurement after sternotomy. It returned to the normal level in the last measurement. This reduction should be seen as a result of the accumulation of total fluid, mainly in the non-fat body mass.

We searched the BMR parameter in other cardiac surgery studies, but it was not found in any of them. In this study, BMR values showed a significant increase compared to preoperative values. This increase in BMR that begins after anesthesia and continues until the end of the operation may seem contradictory with the desired effects of the hypothermia applied, but it shows that the trauma caused by cardiopulmonary bypass cannot be completely prevented despite the retarding effect of hypothermia. It is important in terms of the fact that the patient will need more energy for cell renewal and circulation in the surgical field. This is important for the patient's diet. Phase angle reflects whether the permeability of the cell membrane has changed. This parameter did not change in our study and this shows that there was no change in the cell membrane level. The fact that the total cell mass increased significantly five minutes after the bypass was due to the acceleration of cell production from the bone marrow in response to the reduced body resistance in this stage.

LBM values showed a significant increase in the postoperative period. This increase in LBM values may be the result of an increase in total body fluid and a decrease in FM. The increase in total body mass is due to the increase in total body fluid. We believe there are two reasons for this increase in total body mass. The first is the increase in extracellular fluid during cardiopulmonary bypass. The second is due to the passage of this fluid to the intracellular region after cardiopulmonary bypass and in the early postoperative period.

## CONCLUSION

This study clearly demonstrates that the exchange of body fluids during and after cardiopulmonary bypass is inevitable. In general, the increase in TBW causes an increase in fluid volume in the extracellular compartment during cardiopulmonary bypass. However, in the early postoperative period this increase is shifting to the intracellular region. This may be seen as organ dysfunction due to edema in the organs. Therefore, edema and secondary organ dysfunctions should be closely monitored in the early postoperative period. Increased fluid in the intracellular area should be withdrawn into the vascular bed and appropriate treatment methods should be applied to remove it from the body through diuresis. Also, we believe that BIA, which is a non-invasive procedure, can be safely used in operations where cardiac balance should be closely monitored during and after cardiopulmonary bypass (heart valve patients, low ejection fraction patients, elderly patients, pediatric patients, etc.). Future studies with different patient groups on this subject will increase our knowledge.

**Table:** 

Author's roles & responsibilities	
MG	Substantial contributions to the conception or design of the work; or the acquisition, analysis, or interpretation of data for the work; final approval of the version to be published
CS	Substantial contributions to the conception or design of the work; or the acquisition, analysis, or interpretation of data for the work; final approval of the version to be published
AH	Substantial contributions to the conception or design of the work; or the acquisition, analysis, or interpretation of data for the work; final approval of the version to be published
MSA	Substantial contributions to the conception or design of the work; or the acquisition, analysis, or interpretation of data for the work; final approval of the version to be published
NK	Substantial contributions to the conception or design of the work; or the acquisition, analysis, or interpretation of data for the work; final approval of the version to be published
